# Retraction: Coagulation factor 2 thrombin receptor promotes malignancy in glioma under SOX2 regulation

**DOI:** 10.18632/aging.204295

**Published:** 2022-09-15

**Authors:** Guojun Gao, Ming Yang, Fan Wang, Ge Dang, Xiaoling Zhang, Jing Zhao, Xiangyang Wang, Baozhe Jin

**Affiliations:** 1 Department of Neurosurgery, The First Affiliated Hospital of Xinxiang Medical University, Xinxiang, Henan, PR. China; 2 Department of Neurology, The First Affiliated Hospital of Xinxiang Medical University, Xinxiang, Henan, PR. China; 3 Department of Operating Theatre, The First Affiliated Hospital of Xinxiang Medical University, Xinxiang, Henan, PR China

**This article has been retracted:** Aging has completed its investigation of this paper. We found overlap between some transwell assay images used for Figures 3G and 6E and data previously published by different authors. The authors confirmed the overlap and also raised a concern that the statistical methods used for some analyses were inappropriate. As a result, all of the listed authors agreed that the article should be retracted.

The Administration of the First Affiliated Hospital of Xinxiang Medical University was notified about the retraction by Aging Journal. The Ethics Committee of The First Affiliated Hospital of Xinxiang Medical University agreed that this paper should be retracted because the errors in the statistical methods led to an insufficiently rigorous analysis of the results and because of the improper use of several pictures included in this manuscript. The authors deeply regret any inconvenience this publication has caused for the scientific community.

